# Influence of plasma kinetic energy during the pulsed laser deposition of borophene films on silicon (100)

**DOI:** 10.1039/d3ra04601j

**Published:** 2023-10-11

**Authors:** César D. Rivera-Tello, J. A. Guerrero, L. Huerta, Francisco J. Flores-Ruiz, M. Flores, J. G. Quiñones-Galván

**Affiliations:** a Departamento de Ingeniería Mecánica Eléctrica, CUCEI, Universidad de Guadalajara Blvd. Marcelino García Barragán 1421, Olímpica Guadalajara Jalisco C.P. 44430 Mexico cesar.riveratello@academicos.udg.mx; b Instituto de Investigaciones en Materiales, Universidad Nacional Autónoma de México A. P. 70-360 04510 Ciudad de México Mexico; c CONAHCYT–Instituto de Física, Benemérita Universidad Autónoma de Puebla Ciudad Universitaria, Edif. IF-1 Puebla 72570 México; d Departamento de Ingeniería de Proyectos, Universidad de Guadalajara 45150 Zapopan Jalisco Mexico; e Departamento de Física, CUCEI, Universidad de Guadalajara Blvd. Marcelino García Barragán 1421, Olímpica Guadalajara Jalisco C.P. 44430 Mexico

## Abstract

Developing borophene films with good structural stability on non-metallic substrates to maximize their potential in photosensitivity, gas detection, photothermia, energy storage, and deformation detection, among others has been challenging in recent years. Herein, we succeeded in the pulsed laser deposition of multilayered borophene films on Si (100) with β_12_ or χ_3_ bonding by tuning the mean kinetic energy in the plasma during the deposition process. Raman and X-ray photoelectron spectroscopies confirm β_12_ and χ_3_ bonding in the films. Borophene films with β_12_ bonding were obtained by tuning a high mean kinetic energy in the plasma, while borophene with χ_3_ bonding required a relatively low mean kinetic energy. Atomic force microscopy (AFM) micrographs revealed a granular and directional growth of the multilayered borophene films following the linear atomic terraces from the (100) silicon substrate. AFM nanofriction was used to access the borophene surfaces and to reveal the pull-off force and friction coefficient of the films where the surface oxide showed a significant contribution. To summarize, we show that it is possible to deposit multilayered borophene thin films with different bondings by tuning the mean kinetic energy during pulsed laser deposition. The characterization of the plasma during borophene deposition accompanies our findings, providing support for the changes in kinetic energy.

## Abstract 2

Schematic and idealized illustration of the hexagonal structures of the borophene sheets of the multilayered borophene films; (a) B01, (b) B02, (c) B03, and (d) B04 sample.

## Graphical abstract

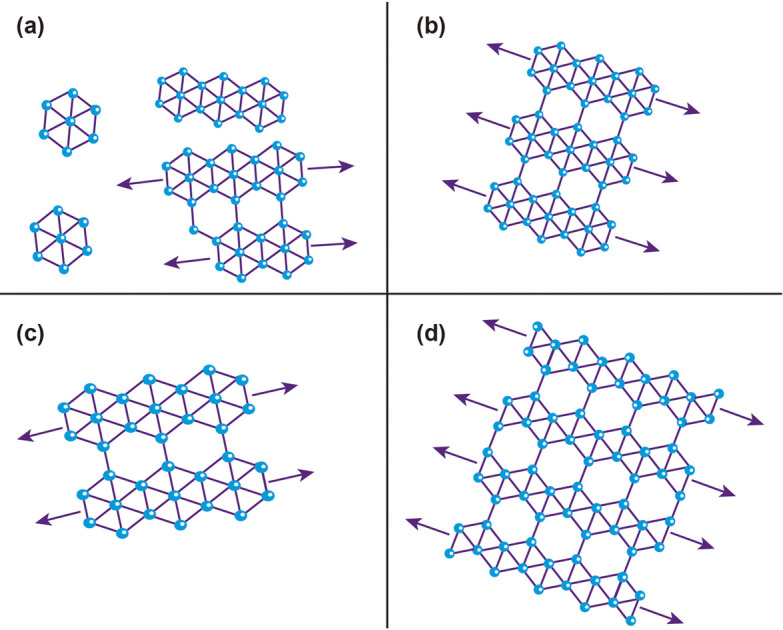

## 1 Introduction

In recent years, the highly metallic behavior of borophene layers has burst into the scene in the development of 2D and nanostructured thin films. They outperform graphene and phosphorene in electrical conductivity applications because they exhibit semi-metallic or semiconducting behavior.^1,2^*Borophene* is a monoatomic thin film of boron atoms arranged in a honeycomb lattice. The high atomic bonding density on the ridgelines of the hexagonal structures of borophene sheets generates an orbital overlap, leading to a high electron density in the conduction levels, which causes highly metallic or superconductor behavior.^3^ Furthermore, the hexagonal holes can act as electron acceptors and boron as donors. Thus, the stability of the monolayer structure depends strongly on the density of the holes, with *η* = 1/9, 1/8, and 2/15 being the most stable.^4–6^ Moreover, anisotropies of electron transport have been reported,^7^ making the physical and electrical properties of borophene layers unique. Another peculiarity of borophene is that it presents different predicted atomic structures conforming to a triangular framework with hexagonal holes (HHs) as a honeycomb lattice. Different kinds of boron monolayer structures (α, β, χ, δ, and ψ) have been proposed according to various possibilities of hexagonal hole densities (*η*) set in the lattice, which conform to 16 confirmed atomic configurations, from *η* = 0 to *η* = 1/3.^8^ However, computational works have predicted that novel phases turn borophene into an extraordinary allotropy compared with other 2D materials.^8–10^ Among these 16 borophene structures, the more stable and well-known ones are β_12_ and χ_3_, which are phases more commonly obtained in experimental processes.^11^ It is worth mentioning that the various hexagonal structures indicate high polymorphism grades on the borophene layers, which could be beneficial in multilayered systems because the structures or bonding tend to change or deform before breaking during the growth process. The borophene polymorphism indicates that different possible structures or bonds with similar formation energy can exist.^11–13^

Boron tends to form bulk structures owing to its electron-deficient property;^14^ therefore, the formation of monoatomic borophene layers became complicated, and its practicality became even more difficult. One way to use borophene monoatomic sheets is by synthesizing or depositing several overlapped borophene sheets to form borophene multilayered films; this term is also known as boron nanosheets.^14^ This growing method allows for more extensive and homogenous areas of borophene multilayered with similar properties to the 2D borophene layers.^15^ Furthermore, the borophene multilayered growth became more significant because the oxidation resistance increased, and there was better chemical and thermal stability than the monolayer growth owing to the formation of covalent B–B bonding in the interlayers.^16^ Moreover, the characterization of these borophene films is accessible because of the higher mass and volume that the spectrometers and microscopies can more easily detect compared to a 2D monoatomic layer. In addition, the deposition processes became more manageable owing to better control of a thicker and more extensive (nanosheets) film.

The three most common plasma deposition techniques used for the growth of borophene layers or sheets are the chemical vapor deposition (CVD), molecular beam epitaxy (MBE), and physical vapor deposition (PVD).^9,17,18^ Among these techniques, the structural bonding obtained depends on the substrate orientation, deposition rate, and kinetics growth.^17^ The PLD is a PVD technique that, when updated with a Langmuir probe, allows access to plasma parameters, such as mean kinetic energy (KE) and plasma density (*N*_p_), which significantly influence the kinetic growth and, consequently, the structural properties of the deposited films. The interaction between the laser and target surface produces non-equilibrium ionized species that reach significantly higher ion energy levels than typical sputtering PVD or CVD techniques. Furthermore, compared with other plasma techniques, PLD generates higher kinetic energies.^19^ In this sense, this technique presents a significant advantage through the control of the plasma parameters because it is possible to obtain high-quality 2D layer materials under vacuum conditions with a deposit process at room temperature (RT). Compared with other deposition techniques, PLD can generate high growth rates and control the morphology and thickness in materials with chemically active surfaces,^20^ such as graphene. Therefore, works related to graphene growth have been intensively developed in recent years.^21–23^ However, borophene layer growth depends strongly on substrate orientation and, more specifically, on atomic terraces. Borophene has been deposited on substrates such as Ag (111),^16^ Al (111),^18^ and Cu (111)^24^ because these metals contain some atomic flat surface areas known as atomic terraces. In all cases, the substrate orientation and the size and number of atomic terraces are key to the obtention of borophene layers. Commonly, the growth of the borophene layer occurs on atomic terraces that are atomically flat, where the boron atoms begin to accommodate themselves in triangular structures to reach the hexagonal structure of the borophene later. Unfortunately, these atomic flat terraces tend to be non-uniform and have irregular growth, especially on metallic substrates,^25,26^ so these fragmented terraces limit the growth of the borophene layers. In this context, recent studies have shown that the semiconductor growth on the directional atomic terraces of the different silicon orientations,^27,28^ such silicon directional atomic terraces are more predictable than those shown in metallic substrates because the atomic terraces from the metallic substrates have irregular size and non-predictable shapes.

Based on the good results obtained in graphene growth by PLD caused mainly by the highly energetic ion species in the plasma of this type of process and taking advantage of both the directional atomic terraces from the silicon (100) substrate and the polymorphism of the borophene layers, we propose the use of the kinetic energy in a PLD plasma to obtain borophene multilayer films. We explore the effect of mean kinetic energy as a control parameter on the structural and chemical composition properties of borophene multilayer films. Kinetic energy control in PLD would be more reliable and reproducible than the traditional parameter of fluence. To the extent we know, there are no reports of borophene or borophene multilayered synthesized by pulsed laser deposition (PLD) on silicon (100), controlling the deposition process by mean kinetic energy.

## 2 Materials and methods

**Fig. 1:**
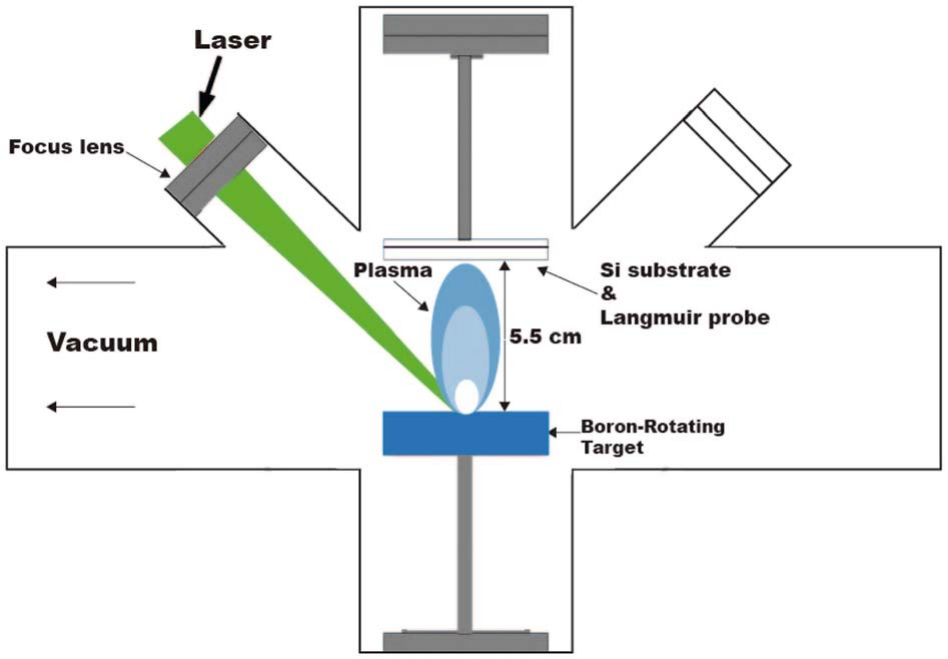
Schematic diagram of the pulsed laser deposition (PLD) system used for the deposition of the four borophene multilayered films.

Four boron films with different mean plasma kinetic energy (KE) values were grown on silicon wafers (100) in vacuum (3.55 × 10^−6^ Torr) by applying the pulsed laser deposition (PLD) technique. A Nd:YAG laser (1064 nm) was used as the fundamental emission line to ablate a boron rotating target surface (99.99% pure) and to generate the plasma plume. Fig. 1 shows the experimental setup of the deposition processes performed at room temperature (RT). The fluence values varied from 2.2 to 7.4 J cm^−2^ to obtain different mean kinetic energies of the boron ions. Fluence was varied by changing the spot size by moving the focusing lens. Table 1 shows the main parameters used to grow the boron films. The PLD processes were controlled by time of flight (TOF) curves obtained by Langmuir-Planar-Probe and one oscilloscope, where the area under the curves was used to estimate the KE; for details of this method, see ref. 29. Prior to each experiment, the TOF signal was measured by placing the probe at the exact position of the substrate using a substrate and probe holder attached to a rotational motion stage and a mask. For plasma measurements, the substrate was placed behind the protected zone of the mask. Afterwards, the probe is moved to place the substrate at the unprotected zone of the mask and start the deposition experiment.

**Table 1:** Ion density and kinetic energy values of the boron film deposition processes obtained from the time of flight (TOF) curves

Sample	Kinetic energy, KE (eV)	Ion density (×10^13^ cm^−3^)
B01	51	2.35
B02	47	2.30
B03	39	2.36
B04	34	2.38

**Fig. 2:**
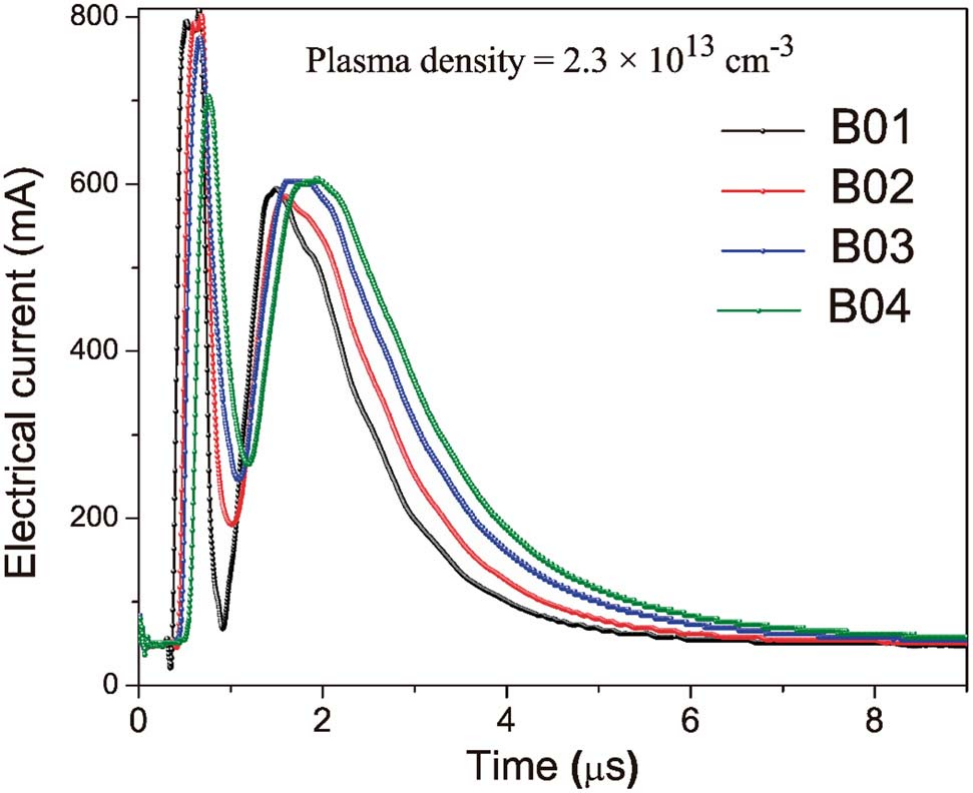
Time of flight (TOF) curves obtained with the Langmuir-Planar-Probe were used for the deposition process of the boron films.

Using mean kinetic plasma energy as a control parameter allows for decreasing error factors associated with fluences, such as spot under focus (changes in spot size), laser incidence angle variations, small pressure changes, laser energy fluctuations due to beam attenuation on windows, and target surface inhomogeneities. All the above factors can modify the incident energy on the target surface, the properties of the generated plasma and, consequently, the growing film properties. The mean kinetic energy of the arriving ionized species highly influences the growing film properties.^29^Fig. 2 shows the Time of Flight (TOF) curves used to control the KE and deposition processes for each boron film. The KE increased with the fluence of the boron films, while the ion density was kept constant. It is worth noting that the intense signal appearing at 0.5 μs for all the samples was not considered in the plasma parameter calculations. This is because this signal is produced by fast ions reaching the probe probably from re-sputtering processes occurring at the probe surface and those they are attracted to and accelerated by the probe electric field. A maximum of the TOF signal can be observed at 1.5 μs, corresponding to the saturation ionic voltage; this value is related to the ion density of the plasma. Additionally, the time at which this saturation voltage occurs is related to the velocity and, thus, the mean kinetic energy of the ions, which means that longer times correspond to particles with lower kinetic energy. This effect of reducing energy can be observed in the TOF curves as a shift of the maximum towards longer times as fluence decreases. The thickness of the boron films was analyzed from cross-sectional images obtained by Field Emission Scanning Electron Microscopy (FESEM) using a TESCAN microscope.

X-ray photoelectron spectroscopy analyses were performed in an ultra-high vacuum (UHV) (Phoibos 150, SPECS) with a monochromatic Al Kα (*hν* = 1486.6 eV) XR50M source. The profiles were made from the surface at 36 min, and the narrow scan sample layer surface was etched for 18 min with 1 kV Ar^+^. The XPS spectra of the B 1s, N 1s, O 1s, and Si 2p core levels were obtained at 45° to the normal surface with constant analyzing energy (CAE) *E*_0_ = 40 eV, 15 eV survey surface and high-resolution narrow scan. The peak positions were referenced to the background Ag 3d_5/2_ photopeak at 367.6 eV, with an FWHM of 0.76 eV, and C 1s hydrocarbon groups at 284.40 eV. The analyzer was corrected by the Atomic Sensitivity Factor (ASF) reported by Scofield^30^ with the reference materials boron, B_2_O_3_, BN, and B + boron oxide by environmental exposure, with an ASF of B 1s = 0.171, N 1s = 0.499, O 1s = 0.733, and Si 2p = 0.368. The spectra were fitted with the program Spectral Data Processor, SDP v4.1.^31^ We found important discrepancies when determining the binding energy (BE) of boron oxidation states at the B 1s core level, similar to those reported by Crist in 2019 and others.^32–35^ The three major problems in all existing data banks are due to (i) the widespread use of different calibration energies, (ii) the widespread use of different energy scales, and (iii) the widespread use of a “user-defined” BE for the C 1s peak attributed to hydrocarbon moieties.^33^ After reviewing scientific articles with similar spectra content, manuals, and digital data banks, we determined to use the average value of B_2_O_3_ from the histograms of NIST BEs for B (1s) BEs, where the average value of B 1s core level is BE = 192.9 eV for B_2_O_3_ based on five literature sources^32^ and elemental boron in 189.4 eV of four sources.^35^ Thus, C 1s corresponds to a BE of 284.4 eV for our samples.

The bonding structure of the films was analyzed by Raman spectroscopy with a Thermo Scientific DXR confocal Raman microscope using the following parameters: wavelength of 532 nm at 5 mW, and an estimated single spot size of 0.5 μm. The background of the spectra was fitted with a Gaussian function. An atomic force microscope (AFM) model, dimension edge bruker, was used to access the surface topography of the borophene films. The same AFM was employed to perform nanofriction tests in a 5 μm × 5 μm area at a scan rate of 1 Hz. The probe had a diamond-like carbon coating [Budgetsensors Multi75DLC, with free resonance frequency of ∼75 kHz and hardness >20 GPa], obtaining AFM topographic surface images and friction force *vs.* load (nN) curves; the procedure for the nanofriction test was reported in ref. 36 and 37.

## 3 Results and discussion

### 3.1 X-Ray photoelectron spectroscopy (XPS)

**Fig. 3:**
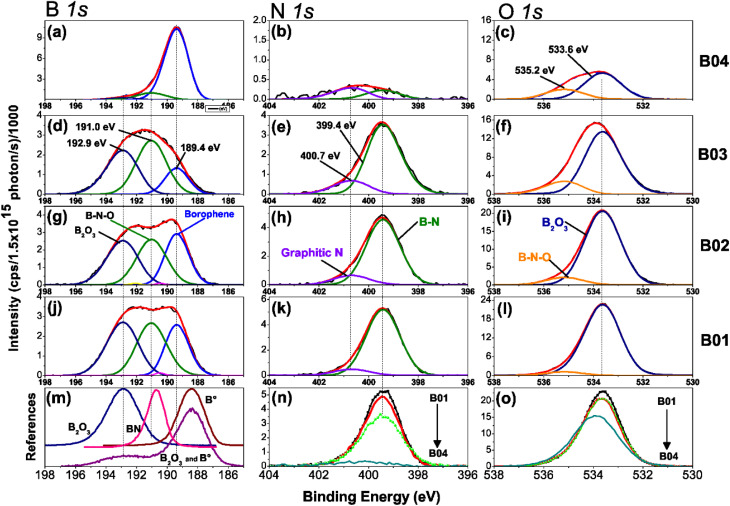
High-resolution XPS spectra of B 1s, N 1s, and O1s core level spectra with their deconvolution analyses of the B01, B02, B03, and B04 samples. The deconvolution analysis of the B 1s orbital is depicted with three contributions of B_2_O_3_, B–N–O, and borophene, as shown in (a), (d), (g) and (j). The N 1s core level with one contribution of B–N and graphitic N is shown in (b), (e), (h), and (k); and the O 1s orbital with two contributions of B_2_O_3_ and BN–O is shown in (c), (f), (i) and (l). Furthermore, the compared reference spectrum of boron of the B 1s orbital is shown in (m); a comparison between the four samples of the N 1s orbital nitrogen content is shown in (n); a comparison of the O 1s core levels of the four samples.

Figure 3(a)–(o) shows the high-resolution XPS spectra of chemical analysis of the boron–boron, boron-oxygen, and boron–nitrogen interaction in borophene multilayered samples; moreover, the high-resolution spectra of the B 1s, N 1s and O 1s core levels are shown. The B 1s peaks are deconvoluted into three components of boron, B–B (lattice boron), B–N–O (BN + O) and B–O (boron oxide), N 1s with B–N and graphitic N signals, and O 1s content two peaks, B_2_O_3_ and B–N–O. Fig. 3(m) shows the reference boron compounds and elemental boron spectra from the XPS libraries.^32^ Applying the corrected C 1s BE peak position spectra value (284.4 eV), the peak position spectra of the B 1s core level at 192.90 eV and 188.40 eV correspond to the B_2_O_3_ compound and elemental boron (B°), respectively. The difference from BE is *Δ* = 4.5 eV, which is the distance between the two chemical states. In our samples, the BE between B_2_O_3_ and borophene is *Δ* = 3.56 eV. Therefore, the borophene peak of our research is present in the spectra B 1s core level (at 189.4 eV) of the four samples [see figures (a), (d), (g) and (j)]. The samples were etched for a few minutes to explore the deep layer profile of the films from 0 to 36 min. After two minutes, the passivated layers of N, C, and O were removed from the surface, leaving stable chemical compounds inside the film.

**Fig. 4:**
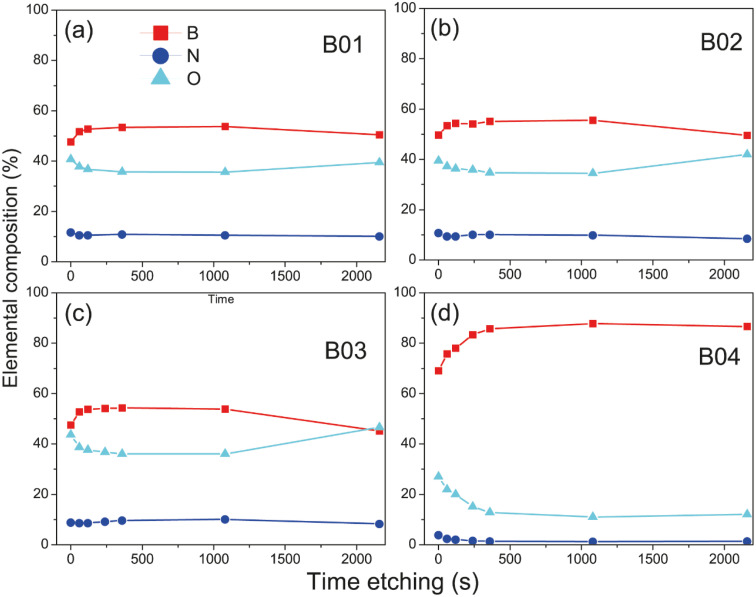
XPS depth profile showing changes in the percentage of elemental composition with the XPS etching time of (a) B01, (b) B02, (c) B03, and (d) B04 films. The depth of the elemental composition increases with time etching. The atomic sensitivity factor (ASF) is used for B 1s.

Deconvolution analysis of B 1s orbitals showed similar borophene spectra (at 189.4 eV) intensities for the B01 and B02 samples, and the B03 sample showed a slight decrease [see Fig. 3(d), (g) and (j)]. However, the peak spectra intensity of the B04 film was significantly higher [see Fig. 3(a)]. In addition, two oxidation states were shown: boron compounds corresponding to B_2_O_3_ with binding energy (BE) at 192.90 eV and the ternary compound of BN + O with BE at 191.04 eV. For the case of B01, B02 and B03, these oxidation states were similar, but for B04, they were significantly lower. This increment and diminution in the quantity of borophene and oxidation states on the B04 film could be related to the molecular structure change, as demonstrated in the Raman section. The N 1s core level spectra have two contributions, corresponding to B–N and graphitic N bonding with BE at 399.42 and 400.69, respectively [see Fig. 3(b), (e), (h) and (k)], and similar peak contributions can be found in the previous works of Chang and coworkers.^38–40^ It is known that the graphitic N bonding formation occurs in the structured lattice and not in altered structures, such as graphene.^41^ A similar behavior is expected for the borophene-graphitic N bonding interactions. Additionally, the O 1s core level also has two peaks at 533.63 eV and 535.17 eV, corresponding to boron oxide and B–N–O contributions, respectively [see Fig. 3(c), (f), (i) and (l)]. Fig. 3(n) and (o) show how the oxidation states of N 1s and O 1s decrease from B01 to B04, respectively. The XPS depth profile results are shown in Fig. 4. The nitrogen is very stable in all samples with boron oxides, where the oxygen inside gradually decreases until it increases around 36 min [see Fig. 4(a)–(c)], except for the B04 sample; in this case, the oxygen content remained constant [see Fig. 4(d)]. This increment could have been generated due to the boron oxides produced at the silicon oxide-boron barrier near the silicon surface when the etching approaches this surface. The B04 profile case confirms what was observed in the B 1s spectra [see Fig. 3(a)], where the borophene and oxidation states increase and decrease, respectively. Elemental boron is not deposited as such from the beginning of the ablation, and it is already different from semimetal boron.

The importance of these XPS results yields the apparition of borophene layers in the four samples, where the B04 sample obtained the highest borophene peak intensity, followed by B02 and B01, and B03, which corresponds to the lowest peak intensity. Higher peak intensity values showed higher borophene bonding in the films, indicating that B04 and B03 had the highest and lowest values, respectively. Moreover, the oxidation states decreased significantly in the B04 sample, suggesting that its bonding energies differed from the rest of the samples. Raman spectroscopy in the next section reveals such bonding differences between B04 and the other three samples.

### 3.2 Raman spectroscopy

**Fig. 5:**
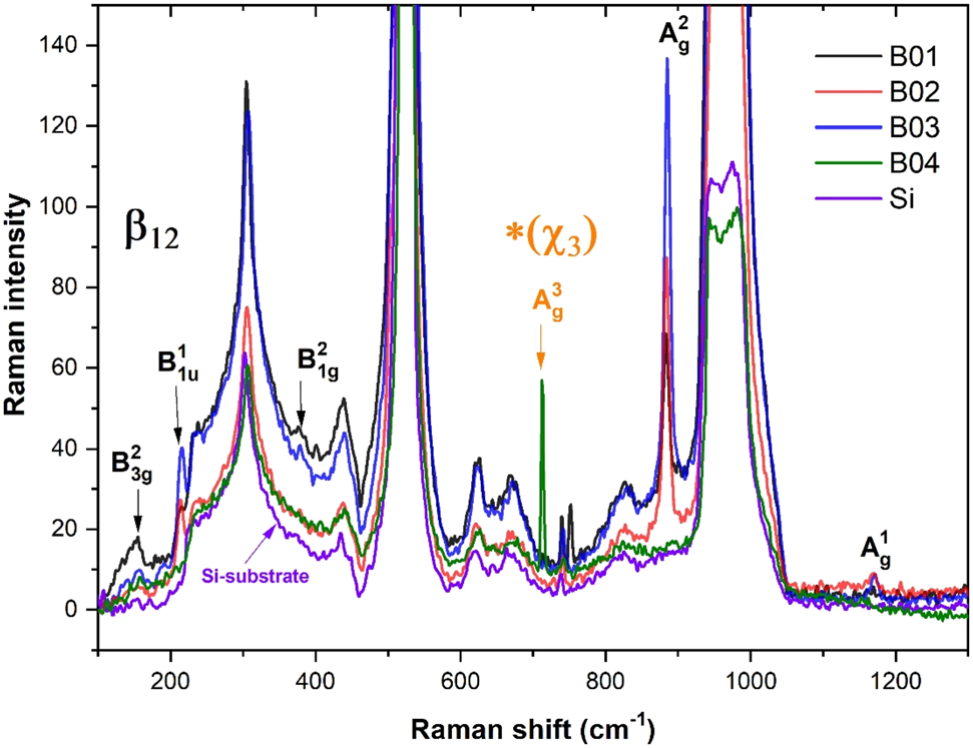
Fitted Raman spectra of the boron films and silicon-substrate corresponding with the vibrational mode values shown in Table 2 were labeled the β_12_ and *χ_3_ bonding.

Fig. 5 shows the Raman spectra taken from the surface of each boron film and silicon substrate. The signal of the silicon substrate significantly influenced the Raman spectra of the films owing to the thin thickness of the samples (27–46 nm; measured by visualizing the cross-section by FESEM). Despite this inconvenience, the typical vibrational modes of β_12_ borophene and χ_3_ (for the case of the B04 sample) were shown in the spectra of the boron films. Table 2 shows the vibrational mode values obtained from our samples compared to the experimental and theoretical values from the study conducted by Sheng *et al.*,^11^ showing similar vibrational mode values, which confirms the presence of β_12_ borophene for B01, B02, and B03 films and χ_3_ for B04. The slight variations in the Raman vibrational position peaks could have been generated owing to the stretched and deformed hexagonal bonding; the hexagonal structures suffered a high polymorphism grade that inevitably altered such a Raman signal. Furthermore, the low Raman signal intensity was due to the overlapping of borophene layers as deposition progressed. Several investigations have shown decrements in the Raman signal when the number of borophene layers increases in borophene films.^42,43^ The highest Raman intensity of the B03 spectrum, especially in the *A*^2^_g_ and *B*^1^_1u_ peaks, indicates the highest number of β_12_ borophene bonds, followed by the B02 and B01 Raman peak intensities. This behavior suggests a direct relation between the KE of the deposition process and the β_12_ borophene bonding; apparently, lower KE values induce the β_12_ borophene bonding. However, for the case of the B04 sample, which was deposited with the lowest KE (34 eV), there is no evidence of the β_12_ borophene bonding, but the presence of χ_3_ bonding is demonstrated, indicating a change in the boron bonding to χ_3_ from 39 to 34 eV. This change can also be seen in the XPS spectrum taken from the B03 sample (see Fig. 3(d)) because the binding intensity of the borophene peak decreased significantly, indicating the possibility of a change in the molecular structure. This could explain the XPS intensity reduction signal from B01 to B03 seen in the borophene peaks. The vibrational modes detected in the Raman spectroscopy increase with the β_12_ bonding because the number of bondings increases, but at the same time, this increment generates more stretched bonding that causes changes in another molecular structure, such as χ_3_. Therefore, when the level of bonding stretched increases, the XPS signal could be reduced; when the changes occurred in the χ_3_ bonding, these stretched levels were reduced, and the XPS signal increased. These stretched bonds were more prominent in the B03 sample than in the other samples, as observed in the AFM section.

**Table 2:** Vibrational modes of the β_12_ bonding from our boron samples compared with the vibrational mode values shown in the study by Sheng^11^

Vibrational modes	Boron samples deposited by PLD (Raman shift, cm^−1^)	Sheng experimental data (cm^−1^)	Sheng theoretical values (cm^−1^)
B^2^_3g_	154	188	186
B^1^_1u_	217	264	255
B^2^_1g_	379	372	417
A^2^_g_	883	856	863
A^1^_g_	1169	1148	1155
A^3^_g_^a^	713	745	769

a Vibrational modes of the χ_3_ borophene bonding.

### 3.3 AFM characterization

**Fig. 6:**
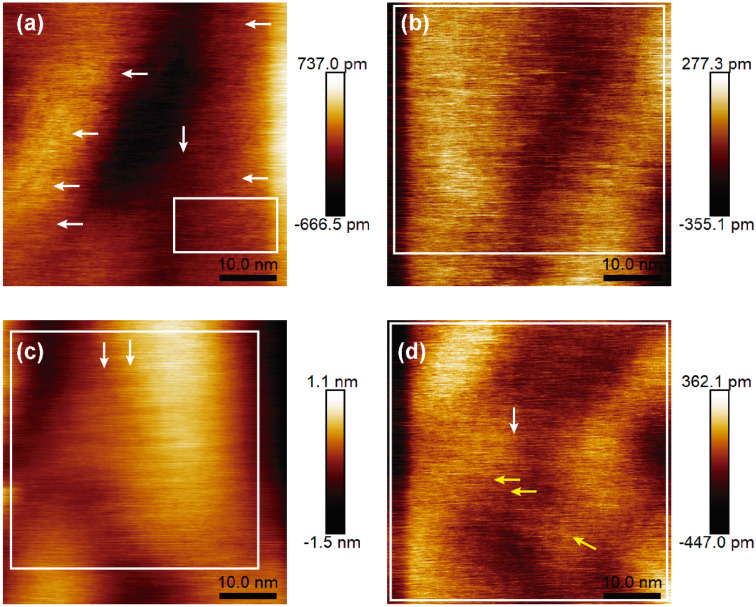
AFM images from the surfaces of (a) B01, (b) B02, (c) B03, and (d) B04 samples. White squares and arrows indicate the most significant hexagonal borophene structures shown. Yellow arrows in (d) indicate thicker ridgelines that could represent half-hexagon from the χ_3_ bonding. The substantial changes in the height and depth of the hills and valleys indicate granular surface morphology.

Figure 6(a)–(d) reveals the surface morphology of the four samples from B01 to B04, respectively. All images show granular growth to a greater extent; above them, some features of hexagonal and polymorphism structures are appreciated, which are representative of borophene sheets. In addition, all the surfaces show directional growth, especially for B02 and B04, where most hexagonal structures followed one determined direction [see Fig. 6(b) and (d)], thereby generating surfaces with uniform and squared appearances. In the case of the B01 surface, the hexagonal structured bonding and the directional growth were less evident, confirming the lowest β_12_ Raman signal and, therefore, the lowest amount of this β_12_ bonding. For the B02 surface, the hexagonal structures were more defined and homogenous, suggesting that the β_12_ bonding increased compared to B01; this can be corroborated with the Raman spectra where the peak of A^2^_g_ increased. The B03 surface showed more prominent and well-defined hexagonal structures in some specific areas [see Fig. 6(c), white arrows]. It seems that the quality and size of this structure were improved compared to those of the B01 and B02 samples; however, the amount of cover area decreased. The highest A^2^_g_ peak intensity showed on this sample in the Raman spectroscopy could corroborate this asseveration (see Fig. 5). The higher stretching level generated by the incoming structural change in this sample could have modified the size and bonding periodicity of the hexagonal structures in this sample. Similar β_12_ structures seen in B01, B02 and B03 are presented in recent previous studies.^11,42^ B04 surface reveals the most defined and uniform hexagonal structures [see Fig. 6(d)]; unlike the B02 surface, B04 shows a better defined hexagonal structure, and it seems that the ridgelines of these structures are thicker compared with the rest of the samples. The larger area of the hexagonal structures and higher uniformity can be corroborated by the highest peak XPS signal intensity of this sample [see Fig. 3(a), (d), (g), and (j)]. In some areas of the B04 surface, it is possible to visualize thicker ridgelines [see yellow arrows in Fig. 6(d)]. Such ridgelines could be half-hexagonal structures from the χ_3_ bonding,^11^ indicating the structural change seen in the Raman spectroscopy section. In all the cases, the granular growth and oxides deformed or stretched and braked some bonding borophene structures, explaining the Raman spectra peak position differences compared to those shown in the referenced work by Sheng.^11^ The direction of the hexagonal structure and the granular growth could have originated from the directional and axial defects of the atomic terraces of the silicon (100) substrate as described below.

### 3.4 Proposed mechanism for the growth of borophene structures on silicon (100)

**Fig. 7:**
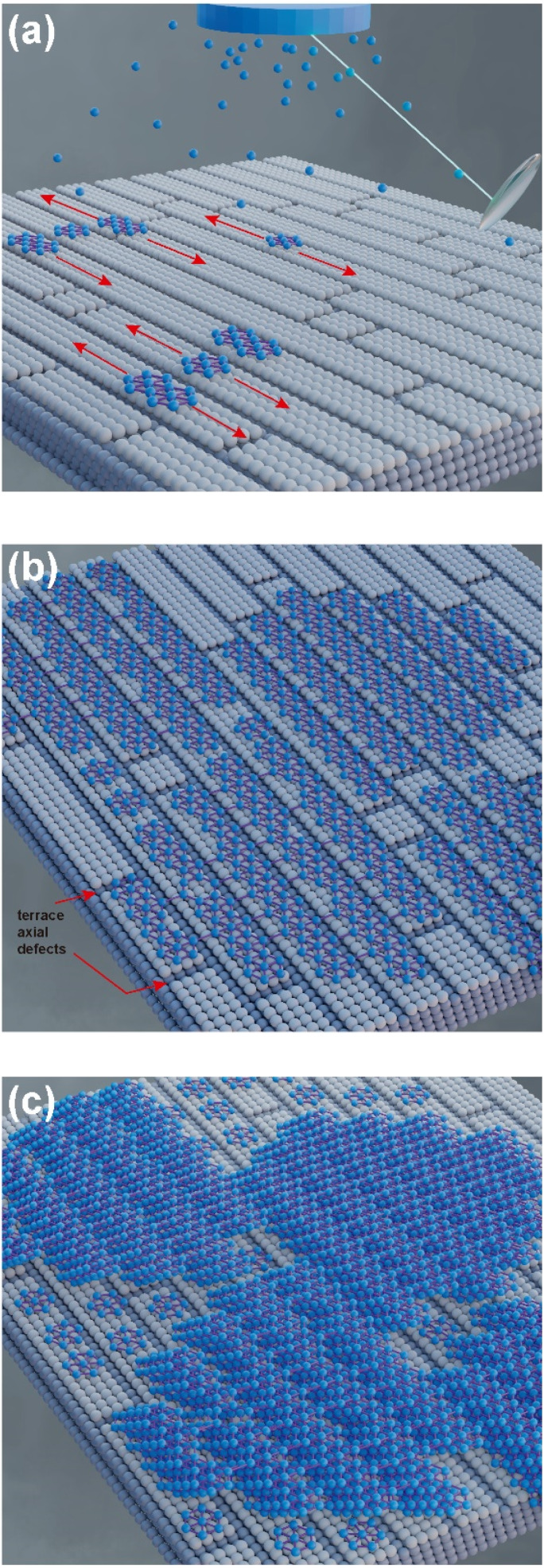
Schematic representation of the chain-granular initial growth of borophene films deposited by pulsed laser deposition: (a) initial growth stage, the first boron-hexagons were formed following the linear silicon terraces; (b) formation of borophene chains cut offered by axial surface defects on the silicon atomic terraces; (c) borophene chain overlapping, forming a multilayered borophene film with granular morphology. White spheres represent silicon atoms, while blue ones represent boron atoms.

**Fig. 8:**
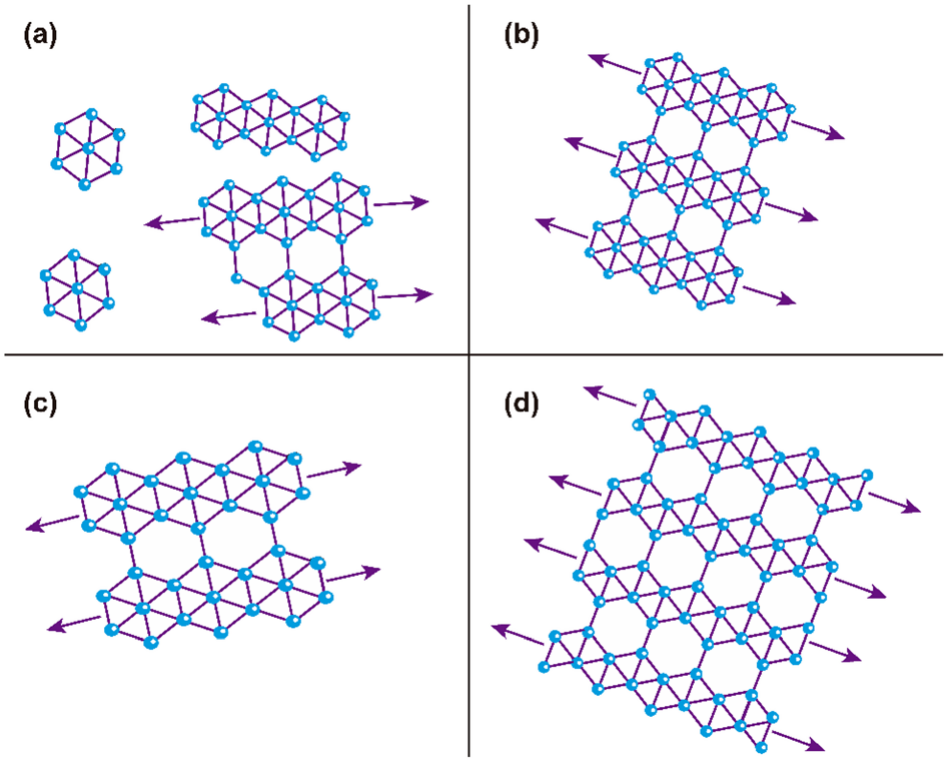
Schematic and idealized illustration of the hexagonal structures of the borophene sheets of the multilayered borophene films: (a) B01, (b) B02, (c) B03, and (d) B04 samples. Arrows indicate directional growth following the silicon 100 atomic terraces. The change in the size of the hexagonal structures of B01 and B03 was in concordance with our experimental evidence, where a slight hexagonal size change was observed. (a), (b), and (c) correspond to the β_12_ and (d) correspond to the χ_3_ bonding detected in Raman spectroscopy.

According to Raman and XPS spectroscopies and some features observed in AFM morphology, we propose a growth mechanism for borophene films on silicon (100), which is schematically displayed in Fig. 7. Fig. 7(a) depicts the initial growth stage, where the boron atoms arrived from the ablation target with high KE levels. Unlike other growth mechanisms reported where the substrate is commonly Cu or Ag, our borophene films do not show diffusion processes. This situation could have originated from the atom size differences between the boron and the substrate atoms (boron atom size: 87 pm; silicon atom size: 111 pm; copper atom size: 145 pm; silver atom size: 165 pm, where data were obtained from ref. 44). A large size of substrate atoms favors interstitial diffusion. In addition, this figure indicates the borophene growth direction, which followed the silicon (100) atomic terraces. Fig. 7(b) illustrates the second growth stage, in which the bonding of the boron atoms with three valence electrons follows the silicon step terraces, forming directional borophene structures. The axial imperfections on the step terraces could have generated granular morphology, cutting the borophene directional growth. Fig. 7(c) depicts the borophene overlapping that started when the Ehrilich-Schwoebel barrier was overcome and started to form the upper borophene layers. In this sense, Wang *et al.*^20^ reported similar growth mechanisms for the case of graphene deposited by PLD. We expect a similar behavior in the case of borophene layer growth owing to the boron and carbon chemical bonding similarities. It is well known that the hexagonal configuration of the borophene layers originates from the three valence electrons, generating triangular and then hexagonal bonding. The atomic terraces promote these bonding mechanisms, so most borophene investigations are usually on Cu (111) and Ag (111). The atomic terraces have larger surfaces, where hexagonal bonding spreads on larger areas. However, 2D imperfections on the terraces break the bonding randomly, making borophene growth unpredictable in most cases. Previous works have shown directional atomic terraces from silicon (100) and other related orientations.^28,45^ In our growth mechanism, we limited these imperfections from the substrate to the 1D direction by cutting only the chain borophene structure and not larger surfaces, as in the case of 2D terraces (Cu and Ag). However, 1D imperfections on the borophene chains, which became axial imperfections, generated granular growth overlapping the borophene layers. This phenomenon results in borophene grain films, which change their 2D properties. Fig. 8(a) to 7(d) shows the schematic and idealized illustration of β_12_ and χ_3_ bonding growth for the B01 to B04 samples, respectively, following the growth model described in Fig. 7 and based on the evidence from the XPS, Raman and AFM characterization.

The evidence from our characterization techniques and analysis suggests that higher energy levels of KE used in the PLD deposition processes generate boron adatoms with excessive motion on the atomic terraces, provoking that they do not completely follow the atomic terraces and form a more aleatory bonding. This situation causes the borophene sheets to overlap less structuredly, such as those shown in the B01 growth. The KE energy decrements generate a lower surface motion to the boron adatoms that allows a more sustainable period on the atomic terraces to start the growth of the borophene structures, following the direction of such terraces, and cut periodically to form, in some cases, the initial growth of a grain. Furthermore, it is worth noting that the surface oxides seen in the XPS section could have generated a more stretched polymorphism in all the samples. In this sense, note that the borophene hexagonal structures were not completely defined in all cases; however, B04 sample showed the more prominent and well defined hexagonal structures and showed the lowest oxide peak content, suggesting a correlation between the oxides and hexagonal-structure-quality.

### 3.5 Nanofriction

**Fig. 9:**
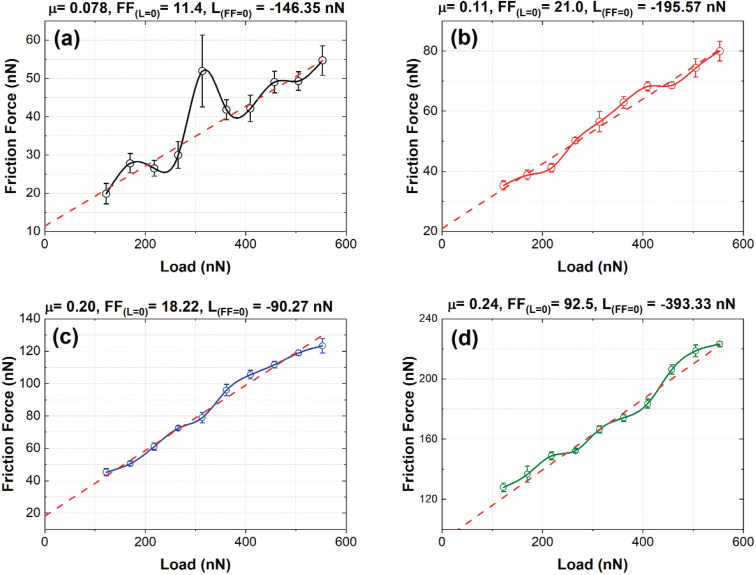
Behavior of the Friction Force compared with the applied load increment of B01 (a), B02 (b), B03 (c) and B04 (d) samples. The graphs also show the friction coefficient (*μ*), the friction force at load = 0, and the pull-off force at FF = 0 (*L*_FF_) values obtained from the data curves.

Fig. 9(a)–(d) shows the friction force (FF) *vs.* load (*L*) graphs obtained from the nanofrictions tests for B01, B02, B03, and B04, respectively. Owing to the oxygen reactivity of the surface, especially for B01 to B03, we considered that the dominant nanofriction mechanism was chemical effects, such as surface chemical reactivity. It is important to remark that other factors influence nanofrictional behavior, such as electrostatic forces, quantity and quality of the mechanic contact and electronic excitations, that could change in the order of the changes on the oxygen reactivity and the borophene type bonding. As noted in the XPS section, the oxides decreased from B01 to B04 [see Fig. 3(o)], where B01 and B04 obtained the highest and lowest quantities of oxides, respectively. However, the B04 showed significantly the lowest oxide amount, and this suggests that the χ_3_ borophene bonding is less oxygen reactive compared to the β_12_ bonding. According to an investigation by Deng *et al.*,^46^ the pull-off force (*L*_FF_) increases in magnitude with exposure to oxygen in a similar bonding structure to borophene, such as graphite. Similar behavior was found in our nanofriction tests; the B01 and B02 surfaces showed higher oxidation levels and high values in the pull-off forces [see Fig. 9(a) and (b)], generating lower friction coefficient values. The case of the B04 sample, which showed the lowest oxidation levels, obtained the highest pull-off force in magnitude [see Fig. 9(d)], showing the highest friction coefficient. According to Deng, this behavior is due to the increment in surface hydrophilicity induced by oxygen chemisorption. However, the B03 sample showed atypical behavior because it had the lowest pull-off force values [see Fig. 9(c)]. This behavior could be explained by observing the XPS O1s spectra [see Fig. 3(f), (i), and (l)], where there was a clear intensity difference between the B_2_O_3_ and B–N–O spectra compared to that of the B01 and B02 spectra. This slight difference could alter the surface hydrophilicity, showing this atypical behavior.

## 4 Conclusions

X-Ray photoelectron and Raman Spectroscopies, as well as the atomic force microscopy analysis, showed that borophene granular multi-layered films with β_12_ and χ_3_ borophene bonding were obtained on the atomic terraces of silicon (100) substrates deposited by pulsed laser deposition, varying the kinetic energy during the deposition process. Both spectroscopies revealed significant changes in their borophene spectra between the samples deposited with higher KE values (51–39 eV) and the sample with a lower value (34 eV), indicating a change in the bonding structure from β_12_ to χ_3_ in the samples with higher and lowest KE values, respectively. AFM micrographs show features related to the influence of directional atomic terraces on the growth mechanism. Such terraces delimited the growth to directional borophene structures, and the axial defects on the silicon atomic terraces could have generated granular growth, stretching, or deforming of the borophene bonding, causing alterations in both spectroscopies used. Moreover, the samples deposited with higher KE energy levels seem to generate excessive motion on the boron adatoms that form a boron bonding growth, which does not follow the silicon atomic terraces, forming less structured borophene sheets. However, lower KE levels could generate more structured formations because of the lower boron motion, which allows for more sustainable growth on atomic terraces. Furthermore, the XPS oxide analysis and the nanofrictional tests revealed that the dominant effect on the friction force curve values was the surface oxidation of the born films. The increments in oxidation generated a pull-off force increment, decreasing the friction coefficients. In addition, such XPS oxide analysis showed that χ_3_ borophene bonding reduces oxidation significantly when compared to β_12_ bonding.

## Conflicts of interest

There are no conflicts to declare.

## Supplementary Material
